# Portal hypertensive gastropathy is associated with iron deficiency anemia

**DOI:** 10.1007/s00508-019-01593-w

**Published:** 2020-01-07

**Authors:** Benedikt Simbrunner, Andrea Beer, Katharina Wöran, Fabian Schmitz, Christian Primas, Marlene Wewalka, Matthias Pinter, Werner Dolak, Bernhard Scheiner, Andreas Puespoek, Michael Trauner, Georg Oberhuber, Mattias Mandorfer, Thomas Reiberger

**Affiliations:** 1 Division of Gastroenterology and Hepatology, Department of Internal Medicine III, Medical University of Vienna, Währinger Gürtel 18–20, 1090 Vienna, Austria; 2 Vienna Hepatic Hemodynamic Laboratory, Medical University of Vienna, Vienna, Austria; 3 Department of Pathology, Medical University of Vienna, Vienna, Austria; 4 Patho im Zentrum, St. Poelten, Austria; 5 Liver Cancer (HCC) Study Group Vienna, Vienna, Austria

**Keywords:** Portal hypertension, Cirrhosis, Anemia, Iron deficiency, Endoscopy

## Abstract

**Background and aims:**

Portal hypertensive gastropathy (PHG) is common in patients with cirrhosis and may cause bleeding. This study systematically explored the independent impact of patient characteristics, portal hypertension and hepatic dysfunction on PHG severity and associated anemia.

**Methods:**

Patients with cirrhosis undergoing endoscopy were included in this retrospective analysis and PHG was endoscopically graded as absent, mild or severe. Clinical and laboratory parameters and hepatic venous pressure gradient (HVPG) were assessed with respect to an association with severity of PHG.

**Results:**

A total of 110 patients (mean age: 57 years, 69% male) with mostly alcoholic liver disease (49%) or viral hepatitis (30%) were included: 15 (13.6%) patients had no PHG, 59 (53.6%) had mild PHG, and 36 (32.7%) had severe PHG. Severe PHG was significantly associated with male sex (83.3% vs. 62.2% in no or mild PHG; *p* = 0.024) and higher Child-Turcotte-Pugh (CTP) stage (CTP-C: 38.9% vs. 27.0% in no or mild PHG; *p* = 0.030), while MELD was similar (*p* = 0.253). Patients with severe PHG had significantly lower hemoglobin values (11.2 ± 0.4 g/dL vs. 12.4 ± 0.2 g/dL; *p* = 0.008) and a higher prevalence of iron-deficiency anemia (IDA: 48.5% vs. 26.9%; *p* = 0.032). Interestingly, HVPG was not significantly higher in severe PHG (median 20 mm Hg) vs. mild PHG (19 mm Hg) and no PHG (18 mm Hg; *p* = 0.252). On multivariate analysis, CTP score (odds ratio, OR: 1.25, 95% confidence interval, CI 1.02–1.53; *p* = 0.033) was independently associated with severe PHG, while only a trend towards an independent association with IDA was observed (OR: 2.28, 95% CI 0.91–5.72; *p* = 0.078).

**Conclusion:**

The CTP score but not HVPG or MELD were risk factors for severe PHG. Importantly, anemia and especially IDA are significantly more common in patients with severe PHG.

**Electronic supplementary material:**

The online version of this article (10.1007/s00508-019-01593-w) contains supplementary material, which is available to authorized users.

The high prevalence of iron-deficiency anemia in patients with cirrhosis indicates the need for awareness and treatment that may improve the quality of life and potentially the outcome of patients with cirrhosis and portal hypertensive gastropathy (PHG). Iron-deficiency anemia and CTP score but not HVPG or MELD were independently associated with severe PHG.

## Introduction

Portal hypertensive gastropathy (PHG) is a common endoscopic finding in patients with cirrhosis and represents a relevant cause of upper gastrointestinal (GI) bleeding [1, 2]. While gastric vascular ectasia (GAVE) may also occur in patients with cirrhosis and lead to GI bleeding, it is a specific gastric mucosal disease entity and differentiation between PHG and GAVE is relevant due to different treatment approaches [2, 3]. In general, PHG is diagnosed via endoscopy and characterized by typical features of the gastric mucosa (i.e. mosaic pattern and red spots). The histology of PHG is characterized by capillary and venous dilation in the gastric mucosal and submucosal layers [3, 4]. The prevalence of PHG in patients with cirrhosis reported by previous studies ranges between 20% and 98% as different definitions and classifications were used and there was also considerable endoscopic and histological interobserver disagreement [4–10].

Chronic anemia is observed in 50–87% of patients with cirrhosis, notably with considerable differences of prevalence with respect to patient population and disease severity [11–14]. Apart from PHG and GAVE, bleeding from gastroesophageal varices and peptic ulcers contribute to anemia in patients with advanced chronic liver disease. While acute or fulminant bleeding from PHG is rare, chronic and subclinical bleeding from PHG is frequent and thus represents an important cause for anemia in cirrhotic patients [3, 6, 7, 15, 16]. Chronic PHG bleeding often leads to microcytic, hypochromic anemia (usually iron-deficiency anemia [IDA]) in 3–26% of patients [3]. Importantly, the presence of anemia in patients with cirrhosis is associated with the occurrence of hepatic encephalopathy, elevated ammonia levels, renal impairment [14], Child-Turcotte-Pugh (CTP) score [12, 13] as well as fatigue and decreased quality of life [17, 18].

Previously reported risk factors for PHG include advanced cirrhosis and previous endoscopic eradication of esophageal varices [4, 19–21]. The grade of PHG was correlated with hepatic venous pressure gradient (HVPG), CTP stage, and Model for End Stage Liver Disease (MELD) [22]. Importantly, patients with severe PHG show a higher relative risk of mortality and have a significantly shorter life expectancy [22]. Treatment of PHG is primarily based on pharmacological treatment aiming to reduce portal pressure as by the use of nonselective betablockers (NSBB) [23–26]. Additionally, PHG considerably improves after transjugular intrahepatic portosystemic shunting (TIPS) [27].

In this exploratory study, we aimed to investigate risk factors for severe PHG in regard to clinical, laboratory and hemodynamic characteristics. In addition, we explored the prevalence and severity of anemia in patients with PHG with particular focus on IDA.

## Patients and methods

### Study design

The study was designed as a retrospective exploratory monocentric cross-sectional study. Laboratory, clinical, hemodynamic (i.e. HVPG), endoscopic and histological data were recorded from patients’ medical records as available. Adult patients (age above 18 years) with an established diagnosis of cirrhosis undergoing upper GI endoscopy were considered for this analysis. Patients after orthotopic liver transplantation, after TIPS implantation and with non-cirrhotic portal hypertension were excluded. Patients received standard of care treatment for portal hypertension according to national and international guidelines, including primary and secondary prophylaxis of variceal bleeding [25, 26, 28].

### Endoscopy and laboratory data

All data were collected from clinical routine testing and endoscopy reports. We assessed vitamin B12 and folic acid levels as deficiencies are commonly found in cirrhosis with or without harmful alcohol consumption. We recorded levels of ferritin and transferrin and transferrin-saturation. Anemia was categorized into mild anemia (Hb <13.5 g/dl in men or <12 g/dl in women), moderate anemia (Hb <10 g/dl) and severe anemia (Hb <8 g/dl). As recently proposed for inflammatory conditions [29, 30], IDA was defined as anemia (Hb levels beneath gender-adjusted lower limit of normal) together with a ferritin concentration <100 µg/L or transferrin saturation <16%. The PHG was classified by experienced endoscopists according to consensus guidelines [31] in three groups: no PHG, mild PHG, and severe.

### Histology

Specimens of the upper GI were obtained during gastroscopy and subsequently fixed in 4% formaldehyde and embedded in paraffin blocks. Microtome cutting and staining with hematoxylin and eosin as well as with modified Giemsa stain was performed by trained technicians and histological analysis of the slides was performed by pathologists trained in gastrointestinal histopathology.

### Statistics

Statistical analyses were performed using IBM SPSS Statistics 25 (IBM, Armonk, NY, USA) and GraphPad Prism 7 (GraphPad Software, La Jolla, CA, USA). Continuous variables are reported as mean ± standard error of the mean (SEM) or median (interquartile range [IQR]), and categorical variables are summarized as numbers (*n*) and proportions (%) of patients. Comparisons of continuous variables were performed using Student’s *t *test or Mann-Whitney U test, as applicable. Categorical variables are presented as numbers and proportions of patients and compared with χ^2^-test or Fisher’s exact test, as applicable. Univariate and multivariate logistic regression analyses were performed to assess risk factors for severe PHG. A two-sided *p*-value ≤0.05 was defined to denote statistical significance.

### Ethics

This study was conducted in accordance with the 1964 Helsinki declaration and its later amendments and approved by the local ethics committee of the Medical University of Vienna (EK1016/2017). Patients consented to the endoscopic procedures. The need for a specific informed consent for this retrospective analysis was waived by the ethics committee of the Medical University of Vienna, since all procedures and tests were performed in clinical routine and only anonymized data are reported.

## Results

### Study cohort

**Fig. 1:**
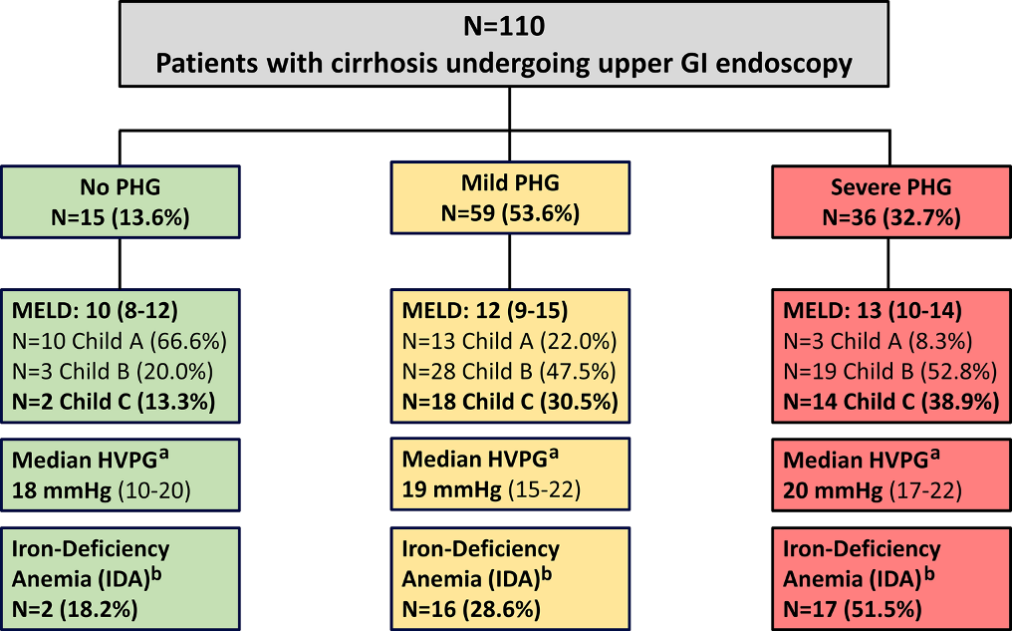
Patient flow chart. ^a^Data on HVPG missing in *n* = 47 (42.7%), available in *n* = 10, *n* = 34, *n* = 19 of patients without PHG, mild and severe PHG, respectively. ^b^Data on IDA missing in *n* = 10 patients (9.1%), available in *n* = 11, *n* = 56, *n* = 33 of patients without PHG, mild and severe PHG, respectively. *GI* gastrointestinal, *PHG* portal hypertensive gastropathy, *MELD* model for end-stage liver disease, *HVPG* hepatic venous pressure gradient, *IDA* iron-deficiency anemia

Among 127 identified patients, 17 had to be excluded due to orthotopic liver transplantation or absence of cirrhosis and/or portal hypertension. Thus, a final number of 110 patients with cirrhosis were included (Fig. 1, Table 1). Most patients were male (69.1%), and alcoholic liver disease (49.1%) and chronic viral hepatitis (30%) were the main etiologies of cirrhosis. Measurement of portal pressure was available in 63 patients and the median HVPG was 19 mm Hg (15–22 mm Hg). Of the patients 30 (27.2%) had a history of previous GI bleeding, of whom 15 had bled from varices and 16 had non-variceal GI bleeding (1 patient had history of both variceal and non-variceal bleeding), 34 (30.9%) patients had previously undergone endoscopic variceal band ligation (EBL).

**Table 1:** Patient characteristics

Patients (*n*, %)	110 (100%)
*Age (years)*	56.8 ± 1.04
*Gender (m/f, % m)*	76/34 (69.1)
Etiology (*n*, %)	
ALD	54 (49.1)
Viral	33 (30.0)
Mixed	8 (7.3)
Other	15 (13.6)
Varices (*n*, %)	
None	37 (33.6)
Small	39 (35.5)
Large	34 (30.9)
Red spots	12 (10.9)
*Prior variceal bleeding (n, %)*	30 (27.2)
*HVPG (mm* *Hg)*^*a*^	18.8 ± 0.74
*Child-Turcotte-Pugh score*	8 (7–10)
*CTP A*	26 (23.6)
*CTP B*	50 (45.5)
*CTP C*	34 (30.9)
*Ascites (n, %)*	
No ascites	38 (34.5)
Mild or medically controlled ascites	41 (37.3)
Severe ascites	31 (28.2)
*Hepatic encephalopathy (n, %)*	
No HE	62 (56.4)
Mild or medically controlled HE	33 (30.0)
Severe HE	15 (13.6)
*MELD*	12 (9–14)
*Platelets (G/L)*	108 (81–158)
*Creatinine*	0.86 (0.72–1.06)
*Bilirubin*	1.38 (0.80–2.39)
*Albumin*	35.0 (29.8–40.2)
*INR*	1.3 (1.2–1.4)
*AST*	38 (23–63)
*ALT*	27 (18–44)
*GGT*	84 (41–150)
*Hemoglobin (g/dL)*	12.0 ± 0.22
*Ferritin*	145.1 (40.5–322.4)
*Transferrin*	232.8 ± 8.38
*TF-saturation (%)*	24.9 (14.8–39.4)
*Iron-deficiency (ferritin <100* *µg/L)*^*b*^* (%)*	40 (43.0%)
*Anemia*	
No anemia	37 (33.6)
Mild anemia (Hb M < 13.5/F < 12g/dL)	52 (47.3)
Moderate anemia (Hb <10g/dL)	16 (14.5)
Severe anemia (Hb <8g/dL)	5 (4.5)
Iron-deficiency anemia (*n*, %)^c^	34 (34.0)

*ALD* alcoholic liver disease, *HVPG* hepatic venous pressure gradient, *HE* hepatic encephalopathy, *MELD* Model for End-Stage Liver Disease, *INR* International normalized ratio, *AST* Aspartate aminotransferase, *ALT* Alanine aminotransferase, *GGT* Gamma-glutamyl transferase, *TF* Transferrin, *Hb* Hemoglobin, *m* male, *f* female

^a^Data on HVPG available in *n* = 63 (57.3%)

^b^No data on iron deficiency in *n* = 17 (15.5%)

^c^No data on iron deficiency-associated anemia in *n* = 10 (9.1%)

### Endoscopy

At upper GI endoscopy, 59 (53.6%) patients had pathognomonic signs of mild PHG and 36 (32.7%) patients were diagnosed with severe PHG (Table 2). Only 15 (13.6%) patients showed no endoscopic signs of PHG, 37 patients (33.6%) had no varices, 39 patients (35.5%) had small varices and 34 patients (30.9%) had large varices. 12 patients (10.9% overall, and 12/73, 16.4% of patients with varices) also showed red spot signs on their varices. The prevalence of varices in patients with severe PHG (26/36, 72.2%) was similar to patients with no (9/15, 60%) or mild (38/59, 64.4%) PHG (*p* = 0.424). Interestingly, the prevalence of severe PHG was not significantly higher in patients who underwent EBL prior to PHG assessment (28.4% for non-severe vs 36.1% for severe PHG, *p* = 0.410).

### Prevalence and severity of PHG according to gender and degree of liver dysfunction and portal hypertension

**Fig. 2:**
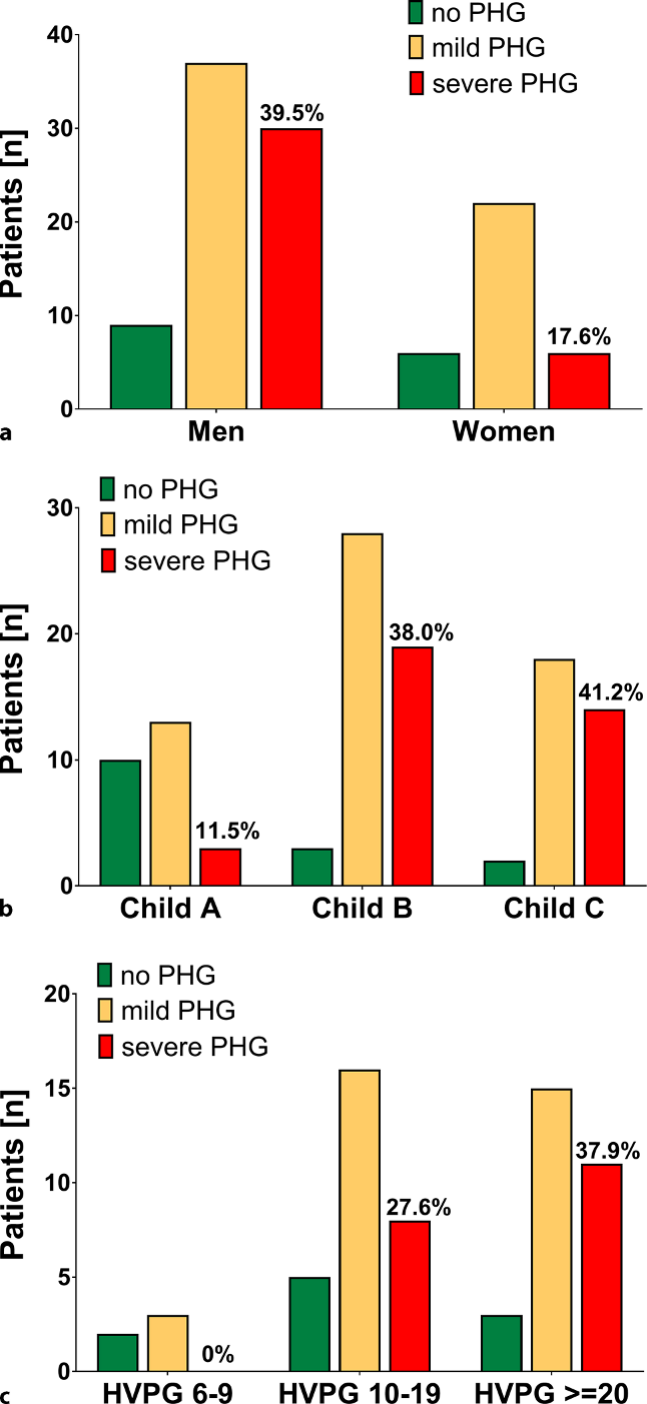
Prevalence and severity of portal hypertensive gastropathy according to **a** gender, **b** Child-Turcotte-Pugh stage and **c** severity of portal hypertension. *PHG* portal hypertensive gastropathy, *HVPG* hepatic venous pressure gradient

We compared characteristics of patients without PHG to patients with mild versus severe PHG. Interestingly, male gender was significantly overrepresented in patients with severe PHG (83.3% male patients vs. 60% with no and 62.7% with mild PHG; *p* = 0.024; Fig. 2, Table 2).

**Table 2:** Comparison of patients without PHG and mild versus severe PHG

	No PHG	Mild PHG	Severe PHG	*p*-value
*Patients (n, %)*	15 (13.6)	59 (53.6)	36 (32.7)	Vs. severe PHG
*Age (years)*	59.4 ± 3.3	57.2 ± 1.4	55.2 ± 1.7	0.272
*Gender (m/f, %m)*	9/6 (60)	37/22 (62.7)	30/6 (83.3)	**0.024**
*Etiology (n, %)*				
ALD	5 (33.3)	29 (49.2)	20 (69.4)	0.745
Viral	7 (46.7)	17 (28.8)	9 (25.0)	
Mixed	1 (6.7)	4 (6.8)	3 (8.3)	
Other	2 (13.3)	9 (15.3)	4 (11.1)	
*Varices (n, %)*				
None	6 (40.0)	21 (35.6)	19 (52.8)	0.424
Small	7 (46.7)	20 (33.9)	12 (33.3)	
Large	2 (13.3)	18 (30.5)	14 (38.9)	
Red spots	0 (0)	7 (11.9)	5 (13.9)	0.523
Prior bleeding	1 (6.7)	7 (11.9)	7 (19.4)	0.244
*HVPG (mm* *Hg)*^*a*^	16.7 ± 2.3	18.7 ± 1.0	20.1 ± 1.2	0.252
*Child-Turcotte-Pugh Score*	6 (6–9)	8 (7–10)	9 (8–11)	**0.018**
*CTP A*	10 (66.6)	13 (22.0)	3 (8.3)	**0.030**
*CTP B*	3 (20.0)	28 (47.5)	19 (52.8)	
*CTP C*	2 (13.3)	18 (30.5)	14 (38.9)	
*MELD*	10 (8–12)	12 (9–15)	13 (10–14)	0.253
*Platelets (G/L)*	117 (91–232)	114 (84–156)	104 (69–145)	0.276
*Hemoglobin (g/dL)*	13.0 ± 0.5	12.2 ± 0.3	11.2 ± 0.4	**0.008**
*MCV (fL)*	87.7 (82.2–96.8)	89.0 (83.9–93.9)	87.7 (81.3–90.0)	0.077
*MCH (pg)*	31.3 (28.8–32.8)	30.7 (29.3–34)	30.4 (28.4–32.1)	0.187
*Ferritin*^*b*^	176.2 (48.2–232.1)	134.8 (28.9–356.8)	158.9 (43.6–294.9)	0.912
*Transferrin*^*c*^	265.8 ± 17.4	231.4 ± 11.0	224.1 ± 16.1	0.477
*Iron deficiency anemia (n, %)*^*d*^	2 (18.2)	16 (28.6)	16 (48.5)	**0.032**
*CRP*	0.36 (0.09–0.98)	0.5 (0.18–1.19)	0.79 (0.5–1.37)	**0.012**
*Histological findings (n, %)*^*e*^				
Capillary ectasia	5 (38.5)	34 (63.0)	23 (67.6)	0.357
Gastritis	11 (84.6)	38 (70.4)	28 (82.4)	0.304
Helicobacter pylori	3 (23.1)	12 (22.2)	9 (26.5)	0.633

*P*-values ≤ 0.05 are indicated in bold

*PHG* Portal-hypertensive gastropathy, *ALD* Alcoholic liver disease, *HVPG* Hepatic venous pressure gradient, *MELD* Model for End-Stage Liver Disease, *CRP* C‑reactive protein

^a^Data on HVPG missing in *n* = 47 (42.7%), available in *n* = 10, *n* = 34, *n* = 19 of patients without PHG, mild and severe PHG, respectively

^b^Data on Ferritin missing in *n* = 17 (15.5%), available in *n* = 10 patients, *n* = 53, and *n* = 30 of patients without PHG, mild and severe PHG, respectively

^c^Data on Transferrin missing in *n* = 18 (16.4%), available in *n* = 10, *n* = 52, and *n* = 30 of patients without PHG, mild and severe PHG, respectively

^d^Data on IDA missing in *n* = 10 patients (9.1%), available in *n* = 11, *n* = 56, *n* = 33 of patients without PHG, mild and severe PHG, respectively

^e^Histological data missing in *n* = 9 patients (8.2%), available in *n* = 13, *n* = 54, *n* = 34 of patients without PHG, mild and severe PHG, respectively

Considering CTP stages, the groups showed the following distribution: in non-PHG patients 66.6% (10 patients) were classified with CTP A, 20.0% CTP B (3 patients) and 13.3% CTP C (2 patients). In contrast, in mild PHG patients only 22.0% were CTP A (13 patients), 47.5% were CTP B (28 patients) and 30.5% (18 patients) were CTP C. Furthermore, in the group of severe PHG only 8.3% (3 patients) were CTP stage A, 52.8% (19 patients) were CTP B and 38.9% (14 patients) were CTP C. The median CTP score was 6 (6–9) in the non-PHG group, 8 (7–10) in the mild PHG group and 9 (8–11) in severe PHG group, and significantly higher in patients with severe PHG as compared to no or mild PHG (*p* = 0.018). Similarly, CTP stage B and C were overrepresented in patients with severe PHG (*p* = 0.03).

Median MELD was comparable among patient groups with respect to the presence of PHG (10 (8–12) points, 12 (9–15) points and 13 (10–14) points among patients without, mild, and severe PHG, respectively; *p* = 0.253).

While non-PHG patients had a median HVPG of 18 mm Hg (10–20), patients with mild PHG had an HVPG of 19 mm Hg (15–22) and patients with severe PHG had 20 mm Hg (17–22). Interestingly, HVPG values were not significantly different among the three patient groups (*p* = 0.252).

### Histological findings in the gastric mucosa of patients with cirrhosis

Histological analysis was performed in 101 patients of our study cohort (Table 3). In the remaining 9 patients, no histological data were available. Gastritis was found in 11 (84.6%) patients without, 38 (70.4%) with mild, and 28 (82.4%) with severe PHG. Prevalence of characteristic histological features for gastritis did not differ between groups (*p* = 0.304). *Helicobacter pylori* was found in a similar proportion across all groups: *H. pylori* was detected in 3 (23.1%) patients without, 12 (22.2%) with mild, 9 (26.5%) with severe PHG (*p* = 0.633). Interestingly, histological features of capillary ectasia were present in similar proportions of patients (*p* = 0.357);however, no quantitative or semi-quantitative scoring of capillary ectasia was performed due to a lack of an established scoring system to assess the severity or grade of capillary ectasia in PHG.

**Table 3:** Independent risk factors for severe portal hypertensive gastropathy (model 1)

	Non-severe PHG	Severe PHG	UVA		MVA	
Patients (*n*, %)	67	33	OR (95%CI)	*p*-value	OR (95%CI)	*p*-value
Age (years)	57.3 ± 1.4	55.2 ± 1.8	0.98 (0.95–1.02)	0.368	–	–
Gender (m/f, % m)	40/27 (59.7)	27/6 (81.8)	**3.04 (1.11–8.34)**	**0.031**	2.76 (0.97–7.85)	0.058
Child-Turcotte-Pugh score	8 (6–10)	9 (8–11)	**1.24 (1.03–1.51)**	**0.026**	**1.25 (1.02–1.53)**	**0.033**
Platelets (G/L)	108 (84–167)	103 (68–145)	1.00 (0.99–1.00)	0.262	–	–
IDA (yes/no, % yes)^a^	18/49 (26.7)	16/17 (48.5)	**2.56 (1.07–6.12)**	**0.034**	2.28 (0.91–5.72)	0.078

*P*-values ≤ 0.05 are indicated in bold

*PHG* Portal-hypertensive gastropathy, *UVA* univariate analysis, *MVA* multivariate analysis, *OR* odds ratio, *IDA* iron-deficiency anemia, *HVPG* hepatic venous pressure gradient, *CI* confidence interval

^a^Data on IDA missing in *n* = 10 patients (9.1%), available in *n* = 11, *n* = 56, *n* = 33 of patients without PHG, mild and severe PHG, respectively

### Independent risk factors for severe PHG

We performed binary logistic regression analyses in order to identify parameters associated with severe PHG (Table 4). Since information on HVPG was only available in a subgroup of patients (*n* = 63, model 2), we also analysed patients with available data on iron deficiency (*n* = 100, model 1) and used platelet counts as a simple surrogate parameter for the severity of portal hypertension.

**Table 4:** Independent risk factors for severe portal hypertensive gastropathy (model 2)

	Non-severe PHG	Severe PHG	UVA		MVA	
Patients (*n*, %)	44	19	OR (95%CI)	*p*-value	OR (95%CI)	*p*-value
Age (years)	56.3 ± 1.8	53.3 ± 2.0	0.98 (0.93–1.02)	0.321	–	–
Gender (m/f, %m)	27/17 (61.4)	16/3 (84.2)	3.36 (0.85–13.3)	0.084	3.84 (0.92–16.0)	0.064
Child-Turcotte-Pugh Score	8 (6–10)	9 (7–11)	1.25 (0.96–1.64)	0.100	1.29 (0.98–1.71)	0.074
HVPG (mm Hg)^a^	18.3 ± 0.9	20.1 ± 1.2	1.06 (0.96–1.16)	0.251	–	–

*PHG* Portal-hypertensive gastropathy, *UVA* univariate analysis, *MVA* multivariate analysis, *OR* odds ratio, *HVPG* hepatic venous pressure gradient

^a^Data on HVPG missing in *n* = 47 (42.7%), available in *n* = 10, *n* = 34, *n* = 19 of patients without PHG, mild and severe PHG, respectively

Male gender (OR 3.04 for male sex, 95% CI 1.11–8.34; *P* = 0.031), CTP score (OR 1.24 per point, 95%CI 1.03–1.51; *p* = 0.026) as well as IDA (OR 2.56, 95%CI 1.07–6.12; *p* = 0.034) were associated with severe PHG on univariate analysis. The CTP score (OR 1.25 per point, 95%CI 1.02–1.53; *p* = 0.033) remained an independent risk factor for severe PHG on multivariate analysis, while only a trend towards an independent association with IDA (OR 2.28, 95%CI 0.91–5.72; *p* = 0.078) and male gender (OR: 2.76, 95%CI 0.97–7.85; *p* = 0.058) was observed.

In the analysis of patients with available HVPG, no parameters where significantly associated with severe PHG, however, despite the small sample size there was still a trend for male sex and CTP score towards an increased risk for severe PHG.

Levels of C‑reactive protein (CRP) were significantly higher in patients with severe PHG (*p* = 0.012); however, CRP levels were not associated with the severity of PHG on binary logistic regression analysis (OR 1.08, 95%CI 0.96–1.21, *p* = 0.189).

### Hemoglobin levels, severity of anemia, and prevalence of iron deficiency anemia according to severity of PHG

**Fig. 3:**
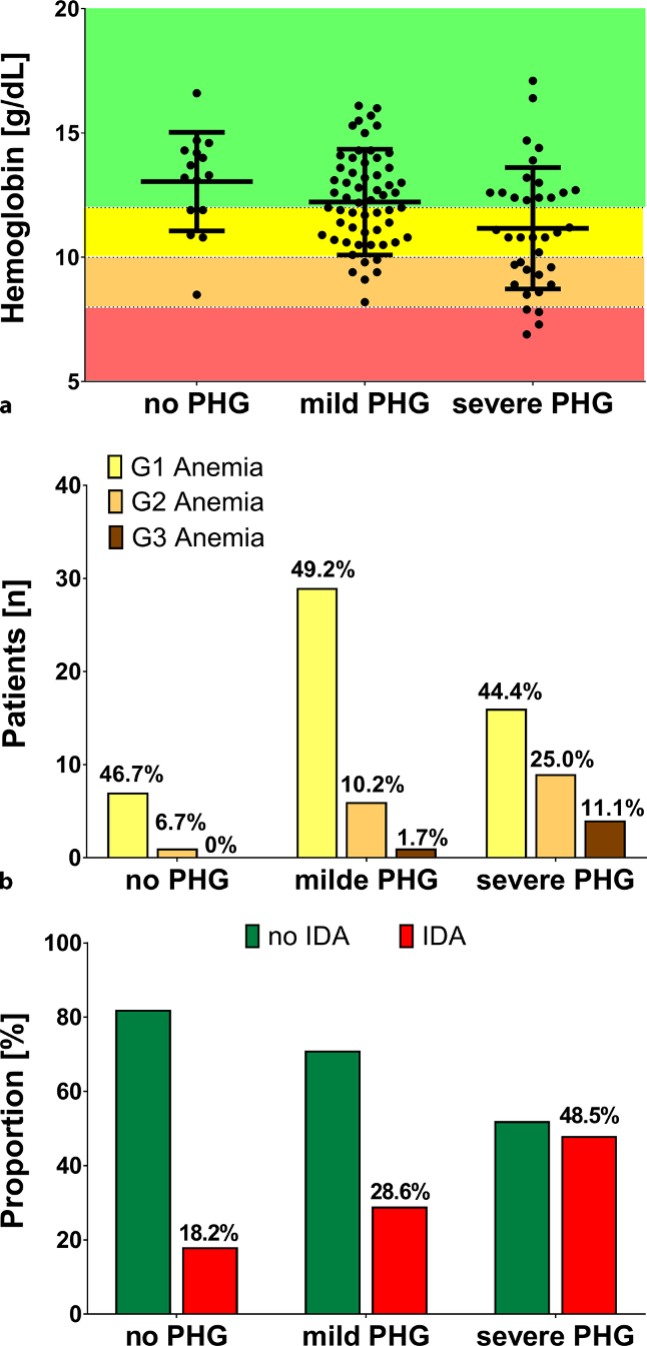
**a** Hemoglobin levels, **b** prevalence of anemia and **c** iron deficiency anemia according to severity of PHG. *PHG* Portal hypertensive gastropathy, *G1–G3* grades 1–3, *IDA* Iron-deficiency anemia

In this study cohort 37 (33.6%) patients had no anemia, while 52 (47.3%) patients presented with mild anemia, 16 (14.5%) with moderate anemia and 5 (4.5%) with severe anemia (Fig. 3). Hemoglobin levels were significantly lower in patients with severe PHG (11.2 ± 0.4 g/dL) versus no PHG (13.0 ± 0.5 g/dL) and mild PHG (12.2 ± 0.3 g/dL; *p* = 0.008; Table 2).

Median values for ferritin were 145.1 µg/L (40.5–322.4), mean transferrin levels were 232.8 ± 8.38 mg/dL and median transferrin-saturation 24.9% (14.8–39.4). Of the patients 40 (43.0%) showed signs of iron deficiency and 34 (34.0%) met criteria for iron-deficiency anemia (IDA). IDA was significantly more prevalent in patients with severe PHG (48.5% vs. 18.2% without PHG and 28.6% with mild PHG; *p* = 0.032). Although MCV (mean corpuscular volume) and MCH (mean corpuscular hemoglobin) were significantly lower in patients with IDA (median MCV 83.7 for IDA vs 90.0, *p* < 0.001; median MCH 28.7 for IDA vs. 31.8, *p* < 0.001; Supplementary Figure S1), most of the patients with IDA did not meet the specific criteria for microcytic and/or hypochromic anemia. Furthermore, MCV and MCH were similar in patients with severe PHG as compared to no or mild PHG (*p* = 0.077 and *p* = 0.187, respectively; Supplementary Figure S2).

## Discussion

In this study, 110 patients (76 men and 34 women) with an established diagnosis of cirrhosis were included in order to assess the presence and/or severity of PHG. Endoscopic diagnosis is usually based on characteristic features of the gastric mucosa, such as mosaic-like and “snake skin” patterns defining mild PHG, and additional red (or “cherry”) spots defining severe PHG that sometimes present with macroscopic signs of chronic bleeding and oozing [3, 31].

Anemia is a common feature in advanced chronic liver disease, affecting up to two thirds of patients with cirrhosis [32]. Anemia was recently associated with increased risk of decompensation and acute-on-chronic liver failure [32]. It is frequently caused by chronic blood loss from gastrointestinal bleeding. Chronic GI bleeding is an important cause of iron-deficiency anemia which is associated with gastrointestinal and liver diseases as well as decreased physical and mental health and even increased mortality [32–34]. The diagnosis of IDA in patients with cirrhosis remains challenging since red blood cell indices, such as MCV and MCH are less reliable due to additional complex vitamin deficiencies and different composition of lipoproteins in erythrocyte membranes [30]. Similarly, while MCV and MCH levels were significantly lower in patients with IDA, most red blood cell indices were within normal range in our study cohort. While ferritin is usually a very reliable marker to measure iron deficiency it also represents an acute phase protein and is upregulated in chronic inflammatory diseases also including chronic liver disease [30]. Thus, the ferritin cut-off level for diagnosis of IDA has been redefined for these diseases [35, 36].

In our study, we detected significantly lower hemoglobin levels in patients with severe PHG. With increased severity of PHG, moderate and severe anemia became more prevalent. Severe anemia has been observed in 11.1% of patients with severe PHG and has also been associated with an increased risk for clinical complications in other cohorts [1]. Overall, IDA was found in about one third of our study cohort. Importantly, almost half of patients with severe PHG were suffering from IDA. This finding clearly supports therapeutic recommendations to supplement iron in patients with PHG suffering from anemia due to chronic bleeding [37]; however, the diagnosis and classification of PHG severity was performed by endoscopy and not by histological criteria. Therefore, the possibility of selection bias in our study cohort must be considered since the medical indication for endoscopy as well as grading of PHG severity is potentially influenced by the concomitance of GI bleeding and, thus, anemia in patients with cirrhosis. The difficulty of reliably assessing the presence of PHG in cirrhotic patients is underlined by the reported prevalence rates ranging between 20–75% in patients with portal hypertension and 35–80% in cirrhotic patients, as displayed in an extensive literature review by Gjeorgjievski and Cappell [38]. These highly heterogeneous findings are most likely explained by differences between study populations and inconsistency of PHG classification [5, 31, 38].

In the present study, higher CTP score as well as more advanced CTP stages were associated with severity of PHG. Moreover, CTP score was an independent risk factor for severe PHG on multivariate analysis. Similarly, although some studies did not find a correlation of CTP stages and the presence or severity of PHG [6, 39], the majority of studies showed a significant association of PHG with the degree of hepatic dysfunction [21, 38, 40, 41]. Previous studies have also shown that duration of liver disease correlates with development of PHG [6, 7]. For example, the proportion of patients with PHG increased by nearly 50% within 5 years of observation [6]. Thus, differing results with respect to PHG and CTP stage in various studies may be in part explained by divergence in duration of disease of included patients and may be additionally dependent on availability and success of etiological treatment such as antiviral therapy for chronic viral hepatitis, as these factors contribute to portal hypertension severity [42, 43].

Furthermore, we assessed whether MELD correlated with the severity of PHG and found no significant difference. Conversely, previous studies showed significant but numerically small differences in MELD score while using similar patients stratification as compared to our study (i.e. no, mild, and severe PHG) [22, 44]. In our study, median MELD increased along the groups but without significant increases of MELD observed in patients with severe PHG.

Interestingly, one previous study reported a mean HVPG of approximately 5 mm Hg in patients without PHG [44], raising the question whether all of the included patients had advanced chronic liver disease; however, it cannot be ruled out that our exploratory study was underpowered to detect a significant difference in MELD between patients with no/mild versus severe PHG. Still it seems that PHG, and even severe PHG can occur at any stage of cirrhosis, and thus already in CTP A patients and in patients with low MELD. Thus, PHG is not just an exclusive feature of advanced stages of chronic liver disease.

In our study male gender was a significant risk factor for severe PHG, which was not reported in previous studies. Interestingly, an experimental study in rodents with portal hypertension suggested that estrogen and progestogen treatment reduces portal pressure, gastric mucosal blood flow, number of blood vessels and relative area of vessels [45]. At this point, however, there is insufficient evidence supporting gender-related endocrinological or hemodynamic influences on PHG. Nevertheless, gender-specific differences in cirrhosis deserve consideration which is underlined by recent studies on patients receiving TIPS [46, 47]. In any case, the association between severity of PHG and male sex deserves to be re-evaluated in prospective patient cohorts.

One of the most controversially discussed topics in portal hypertensive gastropathy is the hemodynamic pathogenesis. Portal hypertension obviously is a key factor in pathogenesis, due to the definition that PHG cannot occur without the presence of portal hypertension [48]. Interestingly, different etiologies of portal hypertension hold a different likelihood to cause PHG, suggesting additional, non-hemodynamic factors in its pathogenesis [21, 49]. In portal hypertension, intrahepatic resistance is increased accompanied by splanchnic vasodilation and decreased systemic vascular resistance [50]. Furthermore, it appears that total gastric blood flow is increased but with decreased mucosal blood flow in the stomach [38]. As reviewed by Perini et al., this hyperdynamic circulation leads to various changes in mediator pathways such as a release of proinflammatory mediators and inhibition of growth factors that potentially promote a susceptibility towards mucosal injury [51].

In our study, we found numerically higher HVPG values with increasing severity of PHG; however, we did not find significant differences of HVPG in patients with severe PHG as compared to patients without or mild PHG. Again, HVPG measurements were only available for 63 of 110 patients, thus statistical power to detect significant differences may not be sufficient. Curvêlo et al. presented similar results, showing no correlation between HVPG and PHG severity [52]. In contrast, a study reporting data from a study cohort of nearly 600 patients found significant differences in portal pressure when comparing portal pressure with respect to the presence/severity of PHG [22]; however, multiple review articles report conflicting data towards the association of PHG with the severity of portal hypertension [3, 38, 51]. In any case, patients receiving TIPS were found to have an amelioration of PHG and a reduced requirement for blood transfusion, thus supporting a beneficial role of therapeutic options aiming at reducing portal pressure for the treatment of PHG [27]. Furthermore, the beneficial effect of HVPG reduction on PHG is further promoted by lower expression of vasoactive proteins in gastric mucosal biopsies in humans upon TIPS [53] and NSBB treatment [54].

Interestingly, CRP levels were higher in patients with severe PHG. Although this does not provide evidence for causality, severe PHG may trigger systemic inflammation. Liver disease and portal hypertension impair intestinal permeability and increase bacterial translocation [55, 56], which in turn may propagate systemic inflammation. Although bacterial translocation may be more prominent in the large and small intestines, severe PHG associated with considerable vascular and mucosal changes may be a valuable biomarker for neovascularization and impaired intestinal permeability in the lower GI, i.e. portal hypertensive enteropathy.

Our retrospective study reports high prevalence of iron-deficiency anemia in patients with cirrhosis. The prevalence of IDA was significantly higher in patients with severe PHG, which is of utmost clinical relevance since awareness and treatment may improve quality of life and potentially the outcomes of patients with cirrhosis and PHG. Interestingly, patient gender was significantly associated with the severity of PHG. Possible mechanisms including the impact of sex hormones on portal and gastric mucosal hemodynamics remain to be assessed in future studies. Interestingly, we did neither identify an association of severity of hepatic dysfunction or of the degree of portal hypertension with PHG severity. Prospective translational studies in humans are warranted to elucidate this controversy. Importantly, critical additional pathogenic factors might be identified from gastric mucosal biopsies obtained from patients with cirrhosis. For example, the prevalence of *H. pylori* infection seems important since specific therapeutic strategies are available and Helicobacter pylori was found in up to 25% of the mucosal biopsies derived from our study cohort.

Prospective studies on PHG with sufficient patient numbers are needed to understand the underlying molecular mechanisms and to explore specific treatment options in patients with PHG.

## Caption Electronic Supplementary Material

Further characterisation of iron-deficiency anemia by analysis of red blood cell indices in patients with cirrhosis.

Further characterisation of iron-deficiency anemia by analysis of red blood cell indices in patients with cirrhosis.
