# Supplementary figures and images for: Portal hypertensive gastropathy is associated with iron deficiency anemia
Source: Wien Klin Wochenschr. 2020 Jan 7;132(1):1–11. doi: 10.1007/s00508-019-01593-w (PMC6978296; doi:10.1007/s00508-019-01593-w)

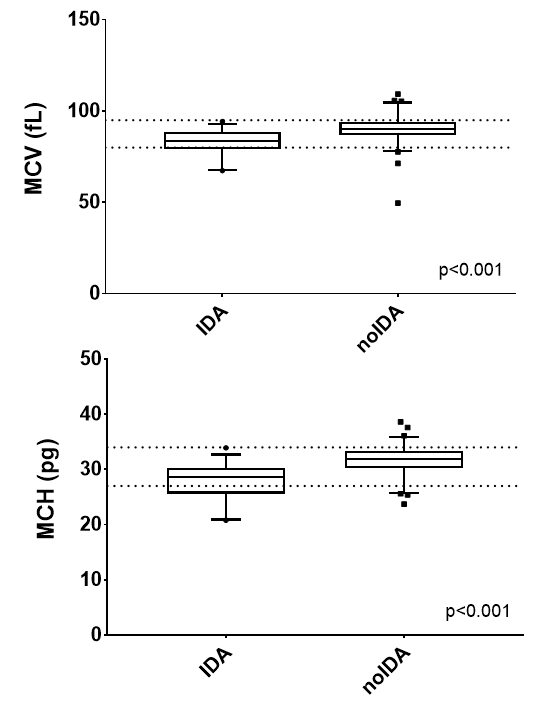

Supplement: Supplementary file 1 — Further characterisation of iron-deficiency anemia by analysis of red blood cell indices in patients with cirrhosis. [file 508_2019_1593_MOESM1_ESM.tif]

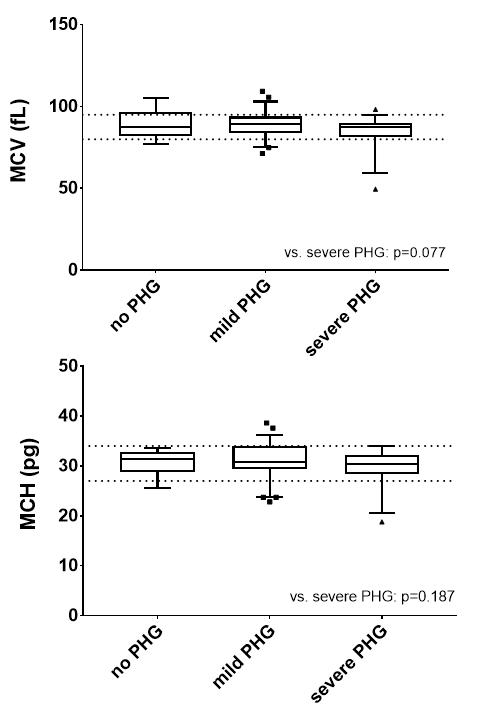

Supplement: Supplementary file 2 — Further characterisation of iron-deficiency anemia by analysis of red blood cell indices in patients with cirrhosis. [file 508_2019_1593_MOESM2_ESM.tif]
